# Ab Initio Study of Ternary W_5_Si_3_ Type TM_5_Sn_2_X Compounds (TM = Nb, Ti and X = Al, Si)

**DOI:** 10.3390/ma12193217

**Published:** 2019-10-01

**Authors:** Ioannis Papadimitriou, Claire Utton, Panos Tsakiropoulos

**Affiliations:** Department of Materials Science and Engineering, The University of Sheffield, Sir Robert Hadfield Building, Mappin Street, Sheffield S1 3JD, England, UK; ioannis.papadimitriou@imdea.org (I.P.); c.utton@sheffield.ac.uk (C.U.)

**Keywords:** ab initio calculations, elastic constants, enthalpies of formation, intermetallics, Nb-silicide based alloys

## Abstract

The adhesion of the scale formed on Nb-silicide based alloys at 1473 K improves when Al and Sn are in synergy with Si and Ti. This improvement is observed when there is segregation of Sn in the microstructure below the alloy/scale interface and a layer rich in intermetallics that include TM_5_Sn_2_X compounds is formed at the interface. Data for the ternary compounds is scarce. In this paper elastic and thermodynamic properties of the Nb_5_Sn_2_Al, Ti_5_Sn_2_Si, Ti_5_Sn_2_Al and Nb_5_Sn_2_Si compounds were studied using the first-principles, pseudopotential plane-wave method based on density functional theory. The enthalpy of formation of the ternary intermetallics was calculated using the quasi-harmonic approximation. The calculations suggest that the Nb_5_Sn_2_Si is the stiffest; that the Nb_5_Sn_2_Al and Ti_5_Sn_2_Si are the most and less ductile phases respectively; and that Nb significantly increases the bulk, shear and elastic moduli of the ternary compound compared with Ti.

## 1. Introduction

The operation of future aero-engines must conform to strict environmental and performance targets. The latter present a challenge to the aerospace industry, and in order to meet them, the turbine entry temperatures (TET) must increase above ~2100 K. In modern aero-engines the TET does not exceed ~1873 K. Ni-based superalloys, which currently are the materials of choice for airfoil applications in gas turbine engines, operate at homologous temperatures approaching 0.8 and pushing them to even higher temperatures is limited by the melting temperature of Ni. Thus, new materials are required to replace Ni-based superalloys. 

New alloys based on refractory metal intermetallic compounds can offer a balance of mechanical and environmental properties [1]. Niobium silicide-based alloys can offer an attractive balance of properties that include oxidation resistance, ballistic resistance, creep, ambient temperature fracture toughness and low and high temperature strength with low density [1]. Their microstructures consist of Nb solid solution and silicide(s) and other intermetallic(s) [2]. The volume fraction of the solid solution critically depends on alloy chemistry and the distribution of phases is dependent on the macrosegregation of Si and other elements. Macrosegregation of solutes can be significant in the cast alloys and depends on alloy chemistry and processing method [3]. 

The oxidation of Nb-silicide based alloys, which was a matter of concern in the early stages of their development, has improved significantly both in the pest oxidation regime and at high temperatures using different alloying strategies [4]. However, the choice of alloying additions and their concentrations in the alloy is restricted by the need to achieve a balance of ambient, intermediate and high temperature properties [1,2]. Improving the adhesion of the scale formed at high temperatures on alloys that do not pest continues to be a challenge. The motivation for the research presented in this paper was to study the properties of one of the phases that can form in the microstructure of the alloy/scale interface at high temperatures. 

Aluminium and Sn improve the oxidation of Nb-silicide based alloys [4,5]. Individually, both elements affect the βNb_5_Si_3_ → αNb_5_Si_3_ transformation and destabilise the tetragonal (stable) Nb_3_Si [6,7,8], and in synergy affect the volume fraction of the Nb_ss_, which can increase or descrease depending on other alloying additions. In general, the concentrations of Al and Sn are restricted to low values (≤5%) because of the adverse effect that these two elements have on toughness. The A15–Nb_3_Sn can be stable in the microstructure of Nb-silicide based alloys depending on the concentration of Sn in the alloy [5]. The impact of each of these two elements on oxidation depends on their concentration and the concentrations of the other elements in the alloy and on the oxidation temperature. Both elements improve the oxidation of Nb-silicide based alloys in the pest regime and Sn is more effective than Al. For example, in isothermal oxidation at 1073 K, the weight gain of the alloy Nb–24Ti–18Si–5Al was higher than that of Nb–24Ti–18Si–5Sn (20.1 and 4.8 mg/cm^2^, respectively). The latter alloy was also more resistant to pest oxidation below 1173 K. Improvement of the oxidation of Nb-silicide based alloys at higher temperatures depends on the synergy of Al and Sn with the other alloying additions. For example, the alloy Nb–24Ti–18Si–5Al gained more weight at 1473 K compared with Nb–24Ti–18Si based alloys with additions of Al, Cr and Ta. The same was the case for Nb–24Ti–18Si based alloys with Sn, where oxidation at high temperatures was improved in the presence of Al, Cr and other transition metal (TM) and refractory metal (RM) elements [9]. Such alloys had lower parabolic oxidation rate constants and better adhering oxide scale than alloys without Sn.

In Nb-silicide based alloys, the microstructure at the alloy (substrate)/scale interface contains contaminated Nb_ss_ (i.e., bcc Nb solid solution with increased solid solubility of oxygen compared with the Nb_ss_ in the bulk of the alloy), and tetragonal Nb_5_Si_3_ silicides with prototype W_5_Si_3_ (tI32, D8_m_) and/or Cr_5_B_3_ (tI32, D8_l_) and hexagonal Nb_5_Si_3_ (hP16, D8_8_, prototype Mn_5_Si_3_) [5]. A15 compounds and other intermetallic phases can also be present [2,5]. In the Nb_5_Si_3_ silicide, the Si can be substituted by Al and Sn [6,7,10]. A15 compounds can be rich in Al, Ge, Si and/or Sn [10]. The A15–Nb_3_Si is a metastable phase in the Nb–Si binary system. The Nb_5_Si_3_ silicide(s) can be contaminated by oxygen, depending on their composition. The A15 compounds also can be contaminated by oxygen.

A characteristic feature of Sn containing Nb-silicide based alloys after oxidation at 1473 K is the segregation of Sn below the substrate/scale interface, which is thought to be responsible for their improved oxidation resistance at this temperature [5,9]. The surface segregation of Sn becomes stronger as the concentration of Sn in the alloy increases, and in the Sn rich microstructure at the substrate/scale interface Nb_3_Sn, Nb_6_Sn_5_ and Nb_5_Sn_2_Si, intermetallics are observed [5,9]. Depending on the concentration of Sn in the alloy, a continuous layer, consisting of some or all of the above intermetallic phases, can form just below the scale. A similar behaviour has not been observed for Al in Nb-silicide based alloys without Sn. However, in alloys where Al and Sn are both present, Al participates in the A15–(Nb,Ti)_3_(Sn,Al), (Nb,Ti)_6_(Sn,Al)_5_ and (Nb,Ti)_5_Sn_2_(Si,Al) intermetallics [5,10]. In such alloys, the adhesion of the scale to the substrate is improved.

The deformation behaviour of the microstructure of the substrate below the oxide scale is one of the factors that determines whether the scale will be retained or spalled off. Thus, knowledge of the properties of the aforementioned phases is essential for improving the oxidation of Nb-silicide based alloys. The properties of unalloyed Nb and tetragonal Nb_5_Si_3_ silicides were recently studied in [11]. The intermetallic phases in the Nb–Sn binary system were studied in [12] and the stability of A15–Nb_3_Sn has been compared with other A15 compounds of interest to the development of Nb-silicide based alloys in [10,13]. There is no data on the mechanical properties of W_5_Si_3_ type TM_5_Sn_2_X compounds. Such data is essential for modelling the deformation of the substrate/scale interface in Nb-silicide based alloys. 

**Figure 1:**
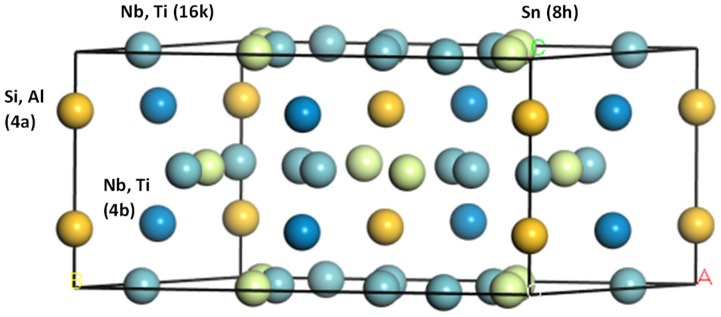
Crystal structure of ternary phases with *tI*32 W_5_Si_3_-type. D8_m_ structure.

The Nb–Sn–Si and Ti–Sn–Si ternary phase diagrams have been reported by Sun et al. [14] and Bulanova et al. [15], respectively. In both ternary systems the existence of a ternary W_5_Si_3_ type compound has been reported, those being the Nb_5_Sn_2_Si and Ti_5_Sn_2_Si for the Nb–Sn–Si and Ti–Sn–Si systems, respectively. The stability of the latter compound was confirmed by Colinet and Tedenac [16]. The first report of the Nb_5_Sn_2_Si compound was by Horyn and Lukaszewicz [17]. Pietzka and Schuster [18] prepared several ternary alloys with a T:M:Al ratio of 5:2:1 where T = Ti, Zr, Hf, V, Nb, Ta, Cr, Mo and W, and M = Si, Ge, Sn and Pb. The Nb_5_Sn_2_Al and Ti_5_Sn_2_Al phases had the same structure. In these phases the T atoms (T = Nb and Ti) occupy the 4b and 16k Wyckoff positions, the Al and Si atoms the 4a positions and the Sn atoms the 8h positions [16,17,18]. Recently the authors reported on the stability of Nb_5_Sn_2_Si in an experimental study of alloys of the Nb–Al–Sn system [19]. The crystal structure for the ternary *tI*32 W_5_Si_3_-type phases is shown in Figure 1.

In the binary phase diagrams of the constituent elements of the aforementioned phases, the high temperature βNb_5_Si_3_ silicide is the only phase that has the same prototype and crystal structure (*tI*32, W_5_Si_3_-type, D8_m_) as the ternary TM_5_Sn_2_X compounds. The Ti_5_Sn_3_ and Ti_5_Si_3_ phases have the *hP*16, Mn_5_Si_3_-type and D8_8_ hexagonal structure, and the Al_5_Ti_3_ has the *tP*32 Ga_5_Ti_3_-type structure. The TM_5_Sn_2_X W_5_Si_3_ type phases do not form only below the scale in the alloy/scale interface of oxidised Nb-silicide based alloys [5] but also in their bulk [19]. 

The present study focused on the TM_5_Sn_2_X (TM = Nb, Ti, X = Al, Si) compounds. Density functional theory (DFT) was used to evaluate the stability and elastic properties and heats of formation of the above ternary intermetallic phases. The data presented in this paper will contribute towards improving the adherence of scales formed on Nb-silicide based alloys and the current understanding of the oxidation and phase equilibria of Nb-silicide based alloys at high temperatures.

## 2. Computational Details

### 2.1. Methodology

First principles calculations were completed using CASTEP (Cambridge Serial Total Energy Package) code [20,21,22] as outlined in [11]. The exchange correlation energy function was estimated by the generalised gradient approximation (GGA) of the Perdew–Wang functional (PW91) [23]. Ultrasoft pseudopotentials [24] were used for ion core and valence electron interactions. The electronic configurations for Nb, Ti, Sn, Al and Si are, respectively, Nb-4s^2^4p^6^4d^4^5s^1^, Ti-3s^2^3p^6^3d^2^4s^2^, Sn-5s^2^5p^2^, Al-3s^2^3p^1^ and Si-3s^2^3p^2^. An energy cut-off of 550 eV was used. Convergence tests showed that it reduced the error in total energy to <1 meV/atom. A Monkhorst–Pack k-point grid of 9 × 9 × 9 for integration over the Brillouin zone was used [25]. Geometry optimisation was performed until the energy change per atom, maximum residual force, maximum atomic displacement and maximum stress were less than 1 × 10^−7^ eV, 1 × 10^−3^ eV/Å, 1 × 10^−4^ Å and 0.001 GPa, respectively [26].

### 2.2. Finite Displacement (Supercell) Method

Finite displacement (supercell) method was used to obtain the phonon DOS for compounds and elements. This method works by calculating the forces on atoms when perturbing the ionic positions [27,28]. The supercell size used for all compounds was 2 × 2 × 2. Convergence tests of the free energy with respect to the cut-off radius were done to achieve an error less than 1 meV/atom. By using the obtained phonon DOS and the formulae in [29], the vibrational contributions to the enthalpy, entropy, free energy and heat capacity versus temperature, along with the Debye temperature, were obtained using the quasi-harmonic approximations.

### 2.3. Elastic Properties

The elastic constants were determined as described in [11]. Simply, a strain was applied and the stress was calculated. The unit cell was fixed and only the internal coordinates were optimised. The matrix of the linear elastic constants was reduced depending on the crystal structure of the phases. For a cubic cell the maximum number of strain patterns (sets of distortions) is one, and for tetragonal or hexagonal cells two patterns are sufficient. Six strain steps (varying from −0.003 to 0.003) were used for each pattern to obtain a consistent linear fit of the stress–strain relationship [11].

To evaluate the six independent elastic constants C_11_, C_12_, C_13_, C_33_, C_44_ and C_66_ for the intermetallic phases, twelve geometry optimisations were done and the mechanical stability criteria checked [30]. From the elastic constants the bulk (B), Young’s (E) and shear (G) moduli and Poisson’s ratio (v) were obtained by using the Voigt–Reuss–Hill (VRH) approximation [31,32]. The Debye temperature at low temperatures was determined from elastic constants using the formulae in [33]. A fit of the energies versus the volumes of the strained structures in the third order Birch–Murnaghan equation of state (B–M EOS) [34] was performed to confirm the values of the bulk moduli.

## 3. Results and Discussion

### 3.1. Elastic Properties

The calculated lattice parameters are shown in Table 1 where they are compared with the values reported in the literature. There is good agreement, as the mean deviation of all the lattice parameters is about 0.6%. The results for the independent elastic constants (C_ij_); bulk moduli (B) from elastic constants, according to the Voigt–Reuss–Hill scheme; and bulk moduli and first pressure derivatives of bulk moduli (B^’^) from the B–M EOS for all intermetallic phases and elements of this study, are shown in Table 2. In Table 3 the calculated values of the shear (G) and Young’s (E) moduli are reported. It was confirmed that the mechanical stability criteria [30] were met for all compounds. The elastic constants were in good agreement with the experimental data for the pure elements [35,36]. To the authors’ knowledge no data exists for the ternary intermetallics. Comparing the bulk moduli obtained from the VRH approximation and the B–M EOS fitting of all the phases, it can be seen that they were in good agreement, with the maximum deviation between them being about 7% for the Ti_5_Sn_2_Al.

The Nb_5_Sn_2_Si phase had the highest values of bulk, shear and Young’s moduli of all the intermetallics, and the Ti_5_Sn_2_Al had the lowest. The sequence was the same for all the moduli; they decreased from Nb_5_Sn_2_Si, to Nb_5_Sn_2_Al, to Ti_5_Sn_2_Si, to Ti_5_Sn_2_Al. It can be seen that all the moduli were significantly higher when Nb was the transition metal in the ternary phase. Furthermore, the Si-containing phases had slightly higher values of bulk, shear and elastic moduli than those that contained Al. 

In a tetragonal phase C_11_, C_12_ and C_33_ correspond to the linear compression resistance along the a, b and c axis. In all cases C_11_ and C_33_ are the highest values. This indicates that linear compression along these axes should be lower. The smallest difference between C_11_ and C_33_ is for the Nb_5_Sn_2_X phases, suggesting that these are less anisotropic than the Ti_5_Sn_2_X phases. If these values are compared with the binary βNb_5_Si_3_ phase [11], which has the D8_m_ structure, the difference between C_11_ and C_33_ is in all cases smaller (between 10 and 30 GPa for ternary phases compared with approximately 60 GPa for βNb_5_Si_3_). In beta Nb_5_Si_3_, the 8 h and 4a Wyckoff positions are filled with Si atoms, whereas in the ternary phase, the 8h position contains Sn atoms. This suggests that the presence of a large, element such as Sn, in the 8h Wyckoff position reduces the anisotropy of the W_5_Si_3_ phase.

To determine whether a material is ductile or brittle, Cauchy pressures (C_12_–C_44_ for cubic and C_13_–C_44_ and C_12_–C_66_ for tetragonal), Pugh’s [40] index of ductility (ratio of shear modulus over bulk modulus (G/B)) and Poisson’s ratio (ν) are taken into account. The values of the aforementioned properties are listed in Table 3. According to Pettifor [41], for metallic bonding, a positive value of Cauchy pressure indicates ductile material and a negative value indicates brittle behaviour. The other two conditions for a compound to be brittle are the G/B ratio to be greater than 0.57 and the ν less than 0.26. Regarding all the aforementioned criteria, the data in Table 3 indicates that the Nb_5_Sn_2_Al and Ti_5_Sn_2_Si phases are the most and less ductile ones, respectively, amongst the intermetallics of the present study. All the ternary intermetallics are more ductile than the βNb_5_Si_3_.

### 3.2. Enthalpies of Formation

**Figure 2:**
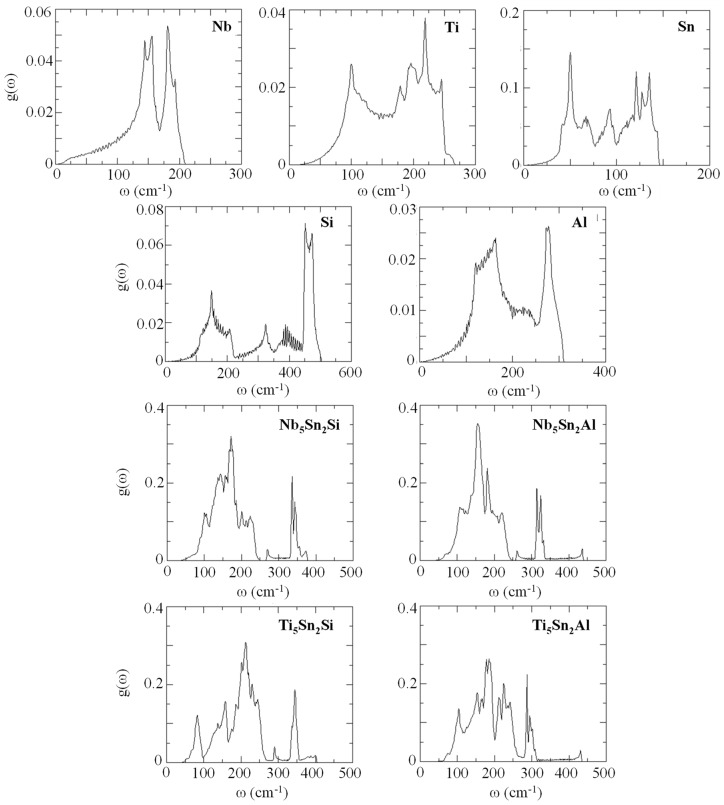
Phonon density of states for Nb, Ti, Sn, Si, Al, Nb_5_Sn_2_Si, Ti_5_Sn_2_Si, Nb_5_Sn_2_Al and Ti_5_Sn_2_Al.

**Figure 3:**
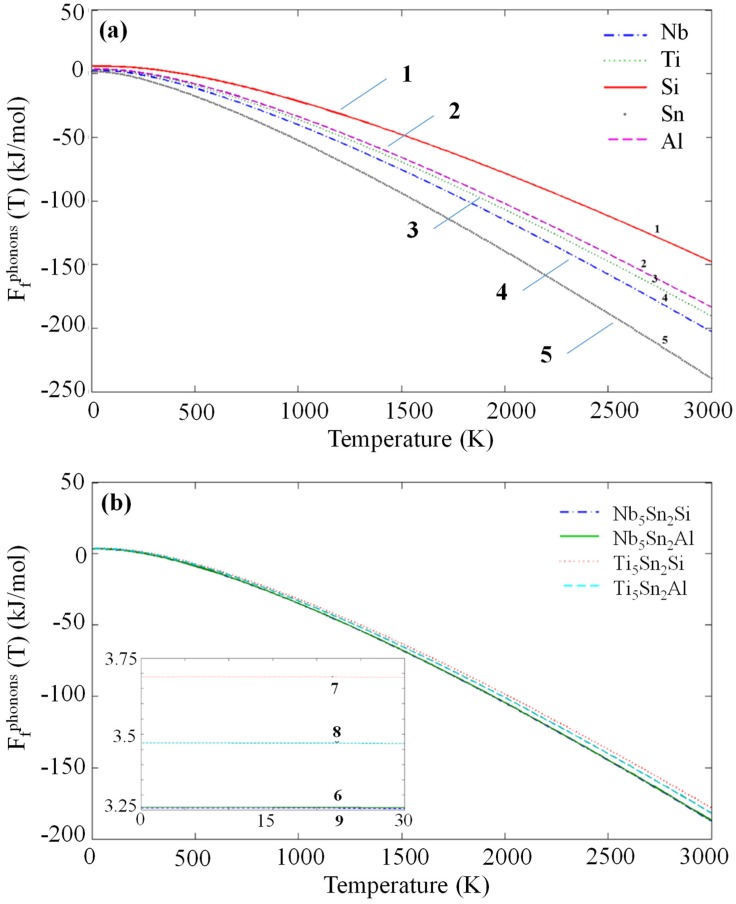
Calculated phonon contribution to free energies for (1) Si, (2) Al, (3) Ti, (4) Nb, (5) Sn, (6) Nb_5_Sn_2_Al, (7) Ti_5_Sn_2_Si, (8) Ti_5_Sn_2_Al and (9) Nb_5_Sn_2_Al.

**Figure 4:**
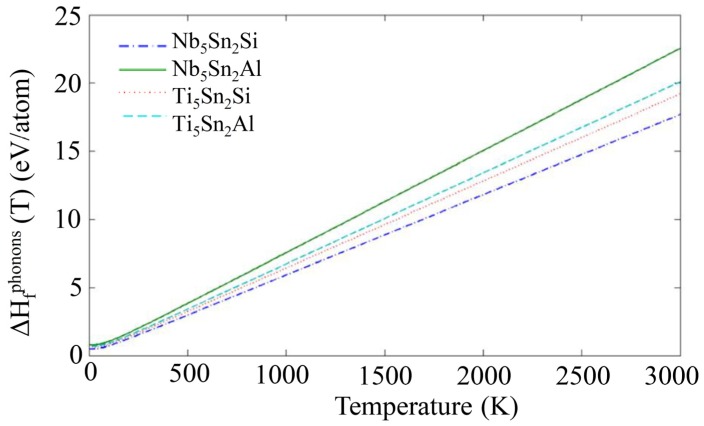
Calculated phonon contributions to enthalpies of formation for Nb_5_Sn_2_Si, Ti_5_Sn_2_Si, Nb_5_Sn_2_Al and Ti_5_Sn_2_Al.

**Figure 5:**
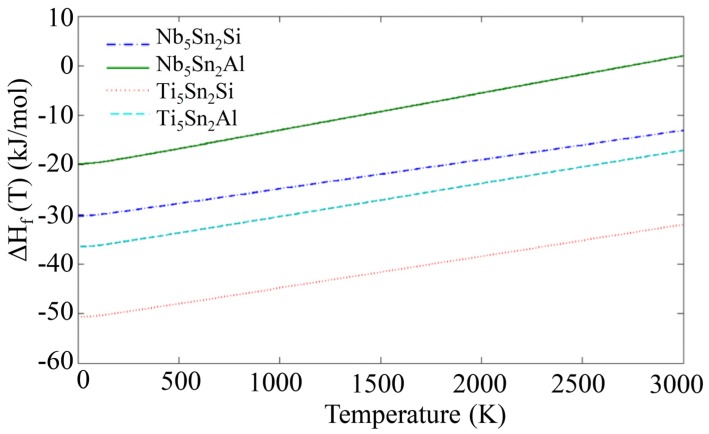
Calculated enthalpies of formation for Nb_5_Sn_2_Si, Ti_5_Sn_2_Si, Nb_5_Sn_2_Al and Ti_5_Sn_2_Al.

The phonon density of states (DOS) for the compounds and elemental phases can be seen in Figure 2. All the eigenfrequencies were found to be real, hence it was confirmed that the compounds under investigation are mechanically stable. After obtaining the computed phonon, DOS the vibrational contribution to free energies per atom (F^phon^ (T)) was calculated (see Figure 3). The F^phonon^ decreased faster in the order: Nb_5_Sn_2_Al, Ti_5_Sn_2_Si, Ti_5_Sn_2_Al and Nb_5_Sn_2_Si. After taking F^phonon^ into account, the phonon contribution to the enthalpy of formation (ΔH_f_^phon^ (T)) was evaluated (see Figure 4). The ΔH_f_^phon^ (T) increased faster for Nb_5_Sn_2_Al. The differences between the ΔH_f_^phon^ (T) of the other three intermetallics were very small. In Figure 5 the enthalpy of formation versus temperature of the ternary intermetallic compounds is shown. As the temperature rises, the ΔH_f_ (T) increases more steeply for the Nb_5_Sn_2_Al, while the curves of the other three phases show approximately the same slope. The data for the heat of formation at T = 0 K, (${\Delta H}_{f}^{0}$), is summarised in Table 4. The (${\Delta H}_{f}^{0}$) increases from Ti_5_Sn_2_Si (−50.655 kJ/mol) to Ti_5_Sn_2_Al (−36.471 kJ/mol) to Nb_5_Sn_2_Si (−30.296 kJ/mol) to Nb_5_Sn_2_Al (−21.516 kJ/mol). Good agreement with available data is shown for Ti_5_Sn_2_Si. 

### 3.3. Debye Temperatures

The resultant phonon DOSs were also used to calculate the Debye temperature. It should be noted that it is considered more difficult to obtain accurate values using this approach than through the elastic constants (see above), because as a low temperature property, the Debye temperature is determined by low energy phonons; i.e., the acoustic phonons. The lower the temperature, the smaller the part of Brillouin Zone that contributes to thermodynamics. The calculated values (Table 3) were in good agreement with [43,46] and the values calculated from elastic constants in the present study. For the elemental phases both the results from the phonon DOS and the elastic constants were in good agreement with the literature. The Nb_5_Sn_2_Si and Nb_5_Sn_2_Al phases had the highest and lowest Debye temperatures, respectively.

## 4. Conclusions

The alloying of Nb-silicide based alloys with Sn increases their oxidation resistance and suppresses pest oxidation, but does not eliminate scale spallation at high temperatures. The synergy of Al and Sn with Si and Ti improves scale adhesion. Oxidation at low and high temperatures is accompanied by the formation of Sn rich areas below the scale at the substrate/scale interface where Sn rich intermetallic phases are formed and Nb_5_Si_3_ is also present. The properties of the former, which include TM_5_Sn_2_X compounds, are important for the retention or spallation of the scale. In this paper, we focused on the Nb_5_Sn_2_Al, Ti_5_Sn_2_Si, Ti_5_Sn_2_Al and Nb_5_Sn_2_Si intermetallics for which data is not available in the literature. The aforementioned compounds and their constituent elements were studied using first-principles calculations. The enthalpy of formation of the intermetallic phases; the elastic constants; bulk (B), shear (G) and Young’s moduli; Poisson’s ratio (ν); and Debye temperature were calculated and are reported for the first time. We used Pugh’s G/B index of ductility, ν and the Cauchy pressures to deduce the ductile or brittle nature of these compounds. Based on our results, the Nb_5_Sn_2_Al and Ti_5_Sn_2_Si compounds are the most and least ductile phases, respectively, and Nb_5_Sn_2_Si is the stiffest and most resistant to deformation. All the ternary intermetallics are more ductile than the βNb_5_Si_3_.

## Figures and Tables

**Table 1:** Lattice parameters (Å) of Nb_5_Sn_2_Si, Ti_5_Sn_2_Si, Nb_5_Sn_2_Al and Ti_5_Sn_2_Al.

Phase	Lattice Parameters	
	a	c
Nb_5_Sn_2_Si	10.683	5.145
-	10.541 [17]	5.138 [17]
Ti_5_Sn_2_Si	10.582	5.05
-	10.558 [16]	5.03 [16]
Nb_5_Sn_2_Al	10.735	5.203
-	10.629 [18]	5.216 [18]
Ti_5_Sn_2_Al	10.612	5.184
-	10.549 [18]	5.242 [18]

**Table 2:** Elastic constants (C_ij_) and bulk modulus (B) for Nb, Si, Al, Sn, Ti, Nb_5_Sn_2_Si, Ti_5_Sn_2_Si, Nb_5_Sn_2_Al and Ti_5_Sn_2_Al in GPa.

Element and Phase	VRH Approximation							B–M EOS	
	C_11_	C_12_	C_13_	C_33_	C_44_	C_66_	B	B	B’
Nb	241	126.3	-	-	26.7	-	164.5	165.1	4.005
-	253 ^a^	133 ^a^	-	-	31 ^a^	-	-	-	-
Si	151.2	57.4	-	-	73.1	-	88.7	91.2	4.009
-	166 ^b^	64 ^b^	-	-	79.6 ^b^	-	-	-	-
Al	107.4	57.6	-	-	30.3	-	74.2	76.47	4.037
-	107 ^b^	61 ^b^	-	-	28 ^b^	-	-	-	-
Sn	74.2	58	22.2	81.2	23.4	9.9	51.8	52.01	3.703
-	72.3 ^c^	59.4 ^c^	35.8 ^c^	88.4 ^c^	22 ^c^	22.5 ^c^	54.9 ^c^	-	-
Ti	149.6 ^d^	97.5 ^d^	79.7 ^d^	186.1 ^d^	33 ^d^	-	110.9 ^d^	118.4 ^d^	4 ^d^
-	160 ^e^	90 ^e^	66 ^e^	181 ^e^	46.5 ^e^	-	-	-	-
Nb_5_Sn_2_Si	303.5	104.4	98.9	313.4	74.4	98.7	169.4	168.8	5
Ti_5_Sn_2_Si	214.8	73.6	71.1	189.6	51.6	75.3	116.6	119.7	5
Nb_5_Sn_2_Al	286.5	97	95.7	269.6	62.5	81.7	157.6	158.6	5
Ti_5_Sn_2_Al	211.5	75.1	63.3	178.6	47.3	69.8	111.1	118.9	5

^a^ Reference [35], ^b^ [36], ^c^ [37], ^d^ [38], ^e^ [39],

**Table 3:** Calculated shear modulus (G) and elastic modulus (E) in GPa; Poisson’s ratio (v), Cauchy pressure (C_12_–C_44_ for cubic and C_13_–C_44_ and C_12_–C_66_ for tetragonal) in GPa; G/B ratio and Debye temperature (θ_D_) from elastic constants; and phonon DOS for Nb, Si, Al, Sn, Ti, Nb_5_Sn_2_Si, Ti_5_Sn_2_Si, Nb_5_Sn_2_Al and Ti_5_Sn_2_Al.

Element and Phase	G	E	-	-	-	-	-	θ_D_ (K)		
	VRH	VRH	v	C_12_–C_44_	C_13_–C_44_	C_12_–C_66_	G/B	Phonon DOS	Elastic Const.	Literature
Nb	36.5	101.9	0.396	99.6	-	-	0.228	277	268	275 ^a^
-	37.5 ^b^	104.9 ^b^	0.397 ^b^	-	-	-	-	-	-	-
Si	61.2	149.2	0.216	−17.4	-	-	0.701	647	628	645 ^a^
-	64.1 ^c^	155.8 ^c^	0.215 ^c^	-	-	-	-	-	-	-
Al	28	74.7	0.334	27.3	-	-	0.377	394	420	428 ^a^
-	26.2 ^b^	70.6 ^b^	0.345 ^b^	-	-	-	-	-	-	-
Sn	16.3	44.3	0.357	-	−1.2	48.1	0.315	254	217	230 ^a^
-	17.7 ^d^	48 ^d^	0.355 ^d^	-	-	-	-	-	-	-
Ti	32.7 ^e^	89.3 ^e^	0.366 ^e^	-	19.5 ^e^		0.295 ^e^	369 ^e^	346 ^e^	380 ^e^
Nb_5_Sn_2_Si	89.7	228.7	0.275	-	24.5	5.7	0.53	311	327	-
Ti_5_Sn_2_Si	61.8	157.6	0.275	-	19.5	−1.7	0.53	305	326	-
Nb_5_Sn_2_Al	77.1	198.9	0.29	-	33.2	15.3	0.489	298	305	-
Ti_5_Sn_2_Al	58.6	149.5	0.276	-	16	5.3	0.527	300	320	-

^a^ [42], ^b^ [43], ^c^ [44], ^d^ Calculated from [37], ^e^ [38,45].

**Table 4:** Enthalpies of formation at T = 0 K for Nb_5_Sn_2_Si, Ti_5_Sn_2_Si, Nb_5_Sn_2_Al and Ti_5_Sn_2_Al.

Intermetallic	Enthalpy of Formation (kJ/mol)	
	Current Study	Literature
Nb_5_Sn_2_Si	−30.296	-
Ti_5_Sn_2_Si	−50.655	−50.751 [16]
Nb_5_Sn_2_Al	−21.516	-
Ti_5_Sn_2_Al	−36.471	-
